# Comparison of experimental ponds for the treatment of dye wastewater under controlled and semi-natural conditions

**DOI:** 10.1007/s11356-017-9245-5

**Published:** 2017-05-23

**Authors:** Dina A. Yaseen, Miklas Scholz

**Affiliations:** 1 Civil Engineering Research Group, School of Computing, Science and Engineering, The University of Salford, Newton Building, Greater Manchester, M5 4WT UK; 2 Division of Water Resources Engineering, Department of Building and Environmental Technology, Faculty of Engineering, Lund University, P.O. Box 118, 22100 Lund, Sweden; 3 Department of Civil Engineering Science, School of Civil Engineering and the Built Environment, University of Johannesburg, Kingsway Campus, PO Box 524, Aukland Park, 2006 Johannesburg, South Africa

**Keywords:** Basic Red 46, Biological treatment, Environmental conditions, Fertiliser, *Lemna minor* L., Pond system, Synthetic dye, Textile

## Abstract

This study compares the performance of simulated shallow ponds vegetated with *Lemna minor* L. under controlled and semi-natural conditions for the treatment of simulated wastewater containing textile dyes. The objectives were to assess the water quality outflow parameters, the potential of *L. minor* concerning the removal of chemical oxygen demand (COD) and four azo dyes (Acid blue 113, reactive blue 198, Direct Orange 46 and Basic Red 46) and the plants’ growth rate. Findings show that all mean outflow values of COD, total dissolved solids (TDS) and electrical conductivity (EC) were significantly (*p* < 0.05) lower within the outdoor compared to the indoor experiment except the dissolved oxygen (DO). The COD removal was low for both experiments. The outflow TDS values were acceptable for all ponds. The pond systems were able to reduce only BR46 significantly (*p* < 0.05) for the tested boundary conditions. Removals under laboratory conditions were better than those for semi-natural environments, indicating the suitability of operating the pond system as a polishing step in warmer regions. The mean outflow values of zinc and copper were below the thresholds set for drinking and irrigation waters and acceptable for *L. minor*. The dyes inhibited the growth of the *L. minor*.

**Electronic supplementary material:**

The online version of this article (doi:10.1007/s11356-017-9245-5) contains supplementary material, which is available to authorized users.

## Introduction

Synthetic dyes are widely used as colouring agents in a variety of technological fields such as the textile, paper production and leather tanning industries (Forgacs et al. 2004) as well as colour photography (Reema et al. 2011). Therefore, the discharge of textile effluents to the environment is undesirable due to the presence of dyes, which are either toxic themselves or often produce toxic breakdown intermediates (Dos Santos et al. 2007). In general, wastewater from textile dye factories has higher biochemical oxygen demand, suspended solids, pH, metals and colour values (Yaseen and Scholz 2016).

The treatment of dyeing effluents has been successfully operated using chemical and physical methods such as oxidation, photocatalysis and ozonation. However, these methods require high costs and involve complicated procedures (Alkhateeb et al. 2005).

Phytoremediation is recommended as one of the cheap biological alternatives for dye removal, which is based on the ability of living organisms such as algae and/or bacteria to clean up pollutants, and the ability of plants to assimilate and accumulate soil, wastewater, water and air contaminants (Sivakumar 2014). Typical processes of wastewater treatment using plants may include degradation, accumulation, dissipation and immobilisation processes of contaminants (Alkhateeb et al. 2005).

Dye degradation in biological treatment processes depends on the chemical structure, molecular weight, as well as the presence or absence of sulpho groups of treated dyes (Yaseen and Scholz 2016) In addition, the operational parameters of the treatment system such as the dye concentration, pH, contact time, adsorbent biomass and temperature also effect the biological dye removal efficiency (Movafeghi et al. 2013).

Phytoremediation techniques have been applied using emergent aquatic plants such as *Phragmites australis* (Cav.) Trin. ex Steud. (Hussein and Scholz 2017) and *Echinodorus cordifolius* L. (Noonpui and Thiravetyan 2011) in constructed wetland systems, and using free-floating plants such as *L. minor* (Uysal et al. 2014) and *Eichhornia crassipes* (Mart.) Solms (Muthunarayanan et al. 2011) in pond treatment systems.

Ponds planted with free-floating macrophytes have been successfully operated for the removal of dyes and other pollutants (Sivakumar 2014; Uysal et al. 2014; Muthunarayanan et al. 2011). However, a great proportion of previous work on the operation of pond systems for dye removal was conducted only for short time periods (few weeks or less). There is also a lack of information concerning the system performance and the main water quality parameters (Yaseen and Scholz 2016). Furthermore, researchers focused on examining artificial ponds for treatment efficiency under either controlled or natural environmental boundary conditions. The performances of identical systems for treating the same dye wastewaters under both semi-natural and controlled conditions have not been previously published. Thus, research is required to focus on comparing the pond system performances and the impact of plants on the dye removal for long time under both controlled and semi-natural conditions.

Among different aquatic plants, *L. minor* has been selected in this study, because this species grows quickly and adapts with no trouble to various aquatic conditions. It is used for the removal of different pollutants and heavy metals from wastewater (Yaseen and Scholz 2016). The study was conducted in Salford, Greater Manchester, UK. Salford is located in North West England (53° 28′ 59″ N, 2° 17′ 35″ W). Environmental conditions are indicated below.

The aim of this investigation is to examine and compare the treatment performance of simulated ponds vegetated with *L. minor* as a polishing stage, which is the last step in a multi-stage system, under controlled laboratory and semi-natural environmental conditions in Salford. The objectives were to assess (i) the water quality parameters including the heavy metals and trace elements; (ii) the potential of *L. minor* on the removal of chemical oxygen demand (COD) and the true colours formed by four azo dyes (Acid blue 113, reactive blue 198, Direct Orange 46 and Basic Red 46) using a spectrophotometric method, which refers to the values as dye concentrations in mg/l; and (iii) the relative growth rate of plants.

## Materials and methodologies

### Dyes and nutrients

The azo dyes reactive blue 198 (RB198), Basic Red 46 (BR46) and Direct Orange 46 (DO46), were provided by Dystar UK Limited (Colne Side Business Park, Huddersfield, UK). Acid blue 113 (AB113) was supplied by Sigma-Aldrich Company UK Limited (The Old Brickyard, New Road, Gillingham, UK), which was used in this study. The chemical formulae and the molecular weights (g/mol) for AB113, RB198, BR46 and DO46 are C_32_H_21_N_5_Na_2_O_6_S_2_, C_41_H_30_C_l4_N_14_Na_4_O_14_S_4_, C_18_H_21_N_6_ and C_12_H_10_N_3_NaO_3_S, and 681.6, 1304.8, 321.4 and 299.2, respectively. AB113 and RB198 belong to the diazo group, whereas BR46 and DO46 are part of the monoazo group.

Stock solutions were prepared for each dye by dissolving 5 g in 1 l of distilled water. The solutions were stored at 4 °C in the dark. The mimicked wastewater was prepared by mixing the dye with an aquatic plant fertiliser called TNC Complete, which was delivered by TNC Limited (Spotland Bridge Mill, Mellor Street, Rochdale, UK). The corresponding nutrient and mineral ingredients were nitrogen (1.5%), phosphorus (0.2%), potassium (5%), magnesium (0.8%), iron (0.08%), manganese (0.018%), copper (0.002%), zinc (0.01%), boron (0.01%) and molybdenum (0.001%). TNC Complete also contained ethylenediaminetetraacetic acid, which comprises copper, iron, manganese and zinc. About 1 ml of fertiliser was mixed with 10 l of dechlorinated tap water. This resulted in a concentration of 5 mg/l per dye. The characteristics of the prepared synthetic wastewater were lower than the typical range (except for colour and pH values) for most textile effluents. This is because the polishing stage is a post-treatment that deals with wastewater already treated in the preliminary and secondary treatment stages. The treatment results in concentrations that are typically lower than those of the textile factory outflows.

### Experiment set-up stages

The study was performed outside on the campus using 58 plastic bowls (length, 33 cm; width, 25.5 cm; depth, 14 cm) as artificial shallow pond simulations: twelve ponds per dye, and ten ponds without dyes were included. The experimental pond system simulates a small part (in terms of width and length) of an artificial shallow and stagnant real pond sealed by an impermeable plastic-based liner and located above ground (directly exposed to the weather).

The containers were filled with dechlorinated tap water to the fill level (depth of 6.9 cm), which is equivalent to 5 l. Thereafter, 200 green and washed *L. minor* plants (about 2.600 ± 0.0292 g) containing four fronds were added to each vessel. Each pond system was supplied with water and fertiliser (see above for composition details) weekly. The plants were obtained from a small pond close to the Cowpe Reservoir (Cowpe, Waterfoot, Lancashire, UK). After finishing the set-up phase, the plants were kept outside to get acclimatised.

**Fig. 1:**
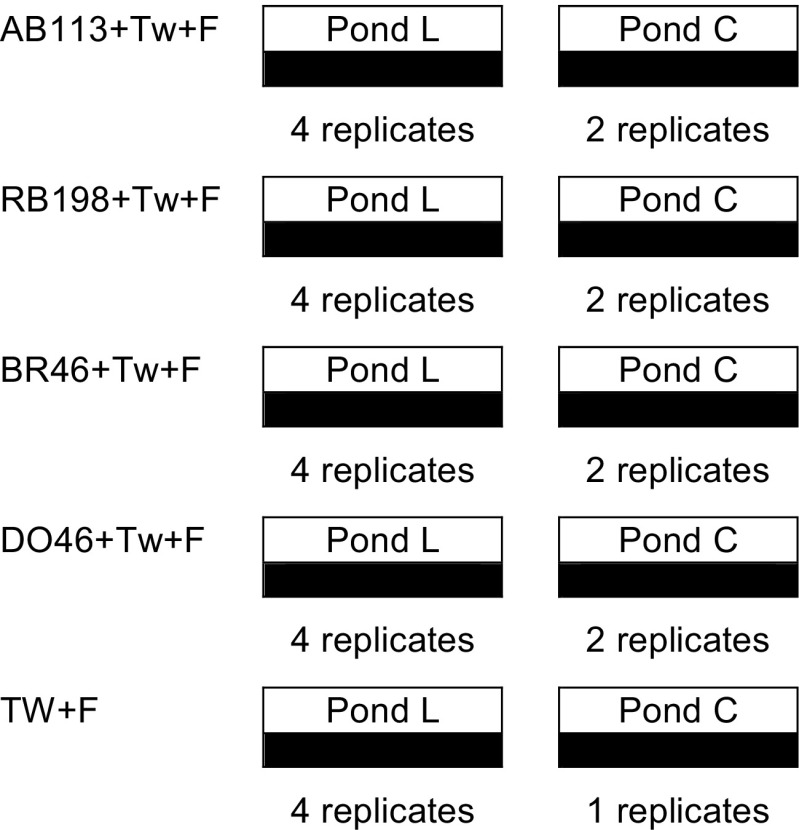
A schematic diagram of the pond set-up (*AB113* Acid blue 113, *RB198* reactive blue 198, *BR46* Basic Red 46, *DO46* Direct Orange 46, *TW+F* tap water and fertiliser, *Pond L Lemna minor* ponds, *Pond C* control ponds)

On 15 December 2014, the main experimental phase began. Twenty-nine containers were located outdoors on the roof of the Newton Building, The University of Salford (Greater Manchester, UK). The set-up comprised of two treatment groups: the first one comprised *L. minor* (L ponds (four replicate)) and the second one denoted the controls without using *L. minor* (C ponds (two replicate)). Further, ponds without dyes were also included. The remaining 29 containers were moved to the nearby Maxwell Building on the same campus, and kept under controlled laboratory conditions using the same set-up (Fig. 1).

The dyes were added to the ponds in this phase on 23 December 2014. All doses had concentrations of 5 mg/l. The corresponding contact time was 7 days. The ponds were stagnant, which means that there was no outflow. Excess water was carefully removed manually. This was required due to occasional high rainfall impacting the outside experiment as mentioned below. However, samples were collected by syringes regularly each week (before adding new dosages) to monitor the system performances. The dosages were added weekly by topping up each pond to the same desired water level equivalent of 5 l as required to address water loss due to evapotranspiration for both set-ups. For the outdoor set-up, sometimes due to the effect of rainfall, the excess water was removed until the level was equivalent to 4 l, and 1 l was added as a new dose in case the water level was higher than the equivalent of 5 l. Note that removal efficiencies were calculated for loads. All water volumes added or removed were considered in these calculations.

### Analysis of water quality

Routine sampling (50 ml) for subsequent water quality assessments (APHA 2005) was performed to monitor system performances. The spectrophotometer DR 2800 Hach Lange (Hach Lange, Willstätter Strasse, Düsseldorf, Germany) was applied for variables including COD, absorbance, apparent colour and suspended solids. Turbidity was measured with a TurbiCheck Turbidity Meter (Tintometer, Lovibond Water Testing, Dortmund, Germany). Both redox potential (redox) and pH were measured with a WTW Vario meter (Cole-Parmer Instrument Co., River Brent Business Park, Trumpers Way, Hanwell, London, UK). Dissolved oxygen was determined with a Hach HQ30d flexi meter (Hach, Pacific Way, Salford, UK). Both electrical conductivity (EC) and total dissolved solids (TDS) were recorded by a METTLER TOLEDO FiveGo™ meter (Keison Products, Chelmsford, UK).

The analysis of dyes was undertaken for filtered water samples of 12 ml each. A 0.45-μm-diameter Whatman filter paper (Scientific Laboratory Suppliers, Wilford Industrial Estate, Ruddington Lane, Wilford, Nottingham, UK) was used. For each dye, the pre-processed water sample was then analysed with a UV-Vis spectrophotometer (DR 2800 Hach Lange) at the maximum absorption wavelengths, which was defined for a solution utilising the UV-Vis spectrophotometer WPA Bio Wave II (Biochrom, Cambourne Business Park, Cambourne, Cambridge, UK). The matching wavelengths were 566, 625, 530 and 421 nm for AB113, RB198, BR46 and DO46, respectively.

Furthermore, metal and trace element analysis was conducted according to EPA (1994) for aqueous samples applying a Varian 720-ES Inductively Coupled Plasma-Optical Emission Spectrometer (Agilent Technologies Wokingham, Berkshire, UK). The samples of 15 ml were filtered using a Whatman filter paper (diameter of 0.45 μm), then acidified using nitric acid S.G. 1.42 (>68%) (Scientific Laboratory Suppliers Ltd.) and preserved in centrifuge tubes at 4 °C.

### Analysis of data

Microsoft Excel (www.microsoft.com) was used for all standard analysis of data, unless stated otherwise. The IBM SPSS Statistics software (version 23) was applied to determine the non-parametric Mann-Whitney *U* test for all data analysis, except for the statistical comparisons among dyes regarding their corresponding outflow concentrations, and dye removal efficiencies, which were analysed using the Kruskal-Wallis test for post hoc comparisons.

### Environmental monitoring

Indoor experiments benefited from OSRAM HQL (MBF-U) High Pressure Mercury Lamp (400 W; base E40) grow lights provided by OSRAM (North Industrial Road, Foshan, Guangdong, China). These laps were supported by a H4000 Gear Unit provided by Philips (London Road, Croyden, UK). Light was controlled by a timer simulating local daylight conditions according to Time and Date (2016).

The mean temperature readings were 27 °C under controlled conditions, and were determined applying the Thermometer-Hygrometer-Station provided by wetterladen24.de (JM Handelspunkt, Geschwend, Germany). However, the mean temperature records under semi-natural conditions were 13 °C. Light recordings were undertaken applying the LUX meter ATP-DT-1300 (TIMSTAR, Road Three, Winsford Industrial Estate, Winsford, UK). Measurements were taken directly above the plants, which were 2215 lx under controlled conditions and 16,507 lx under semi-natural conditions. The mean values of environmental conditions for Salford (outside conditions) and the laboratory (inside conditions) during the study period are presented in Table 1.

**Table 1:** Mean values of parameters characterising environmental conditions

Condition	Parameter	Unit	Mean	Standard deviation	Minimum	Maximum	Number of records
Laboratory	Temperature	°C	27.3	2.59	22.4	29.9	127
	Illuminance	lux	2215.4	145.52	2023	2450	33
	Relative humidity	%	53.7	4.08	36.0	60.0	127
Semi-natural	Temperature	°C	12.6	7.3	−3.0	29.0	180
	Illuminance	lux	16,506.6	16,143.7	2010	49,675	35

## Results and discussion

### Water quality analysis

**Fig. 2:**
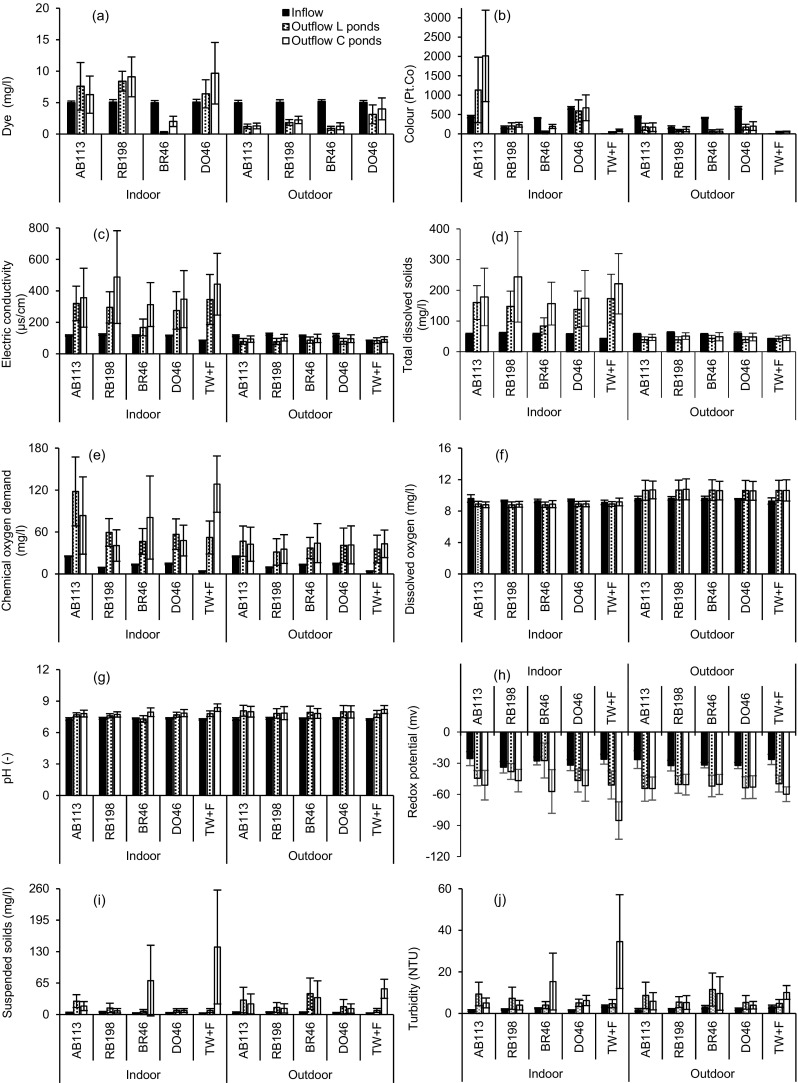
Mean and standard deviation of inflow and outflow water characteristics for the indoor and outdoor experiments between 15 December 2014 and 15 September 2015 (*AB113* Acid blue 113, *RB198* reactive blue 198, *BR46* Basic Red 46, *DO46* Direct Orange 46, *TW+F* tap water and fertiliser; (**a**) dye concentration, (**b**) colour, (**c**) electric conductivity, (**d**) total dissolved solids, (**e**) chemical oxygen demand, (**f**) dissolved oxygen, (**g**) pH, (**h**) redox potential, (**i**) suspended solids, (**j**) turbidity)

The mean values for inflow and outflow water characteristics regarding the indoor and outdoor experiments between 15 December 2014 and 15 September 2015 (study period) are shown in Fig. 2. The average inflow water quality parameters for the outdoor experiment compare with those for the indoor one; no significant (*p* > 0.05) differences were noted between the mean values for all water quality parameters between the two experiments.

### Dye concentrations and colour

The average outflow dye values concerning the *L. minor* and control pond systems were significantly (*p* < 0.05) higher for the indoor experiment (see Fig. 2a) than the outdoor one for all dyes, except for *L. minor* ponds comprising BR46. The mean outflow BR46 values for planted ponds were significantly (*p* < 0.05) lower within indoor experiments than the outdoor ones, which were 0.3 and 0.9 mg/l for the indoor and outdoor experiment, respectively. If compared to the inflow dye concentration of 5 mg/l, this was evidence due to the high degradation of the dye BR46 under controlled conditions compared to other studied dyes, as indicated later in the “Dye removal” section. Overall, the outflow BR46 concentrations in planted ponds were significantly (*p* < 0.05) reduced compared to the corresponding outflow concentrations for the other dyes under both environmental conditions.

All colour mean outflow values (as illustrated in Fig. 2b) were significantly higher (*p* < 0.05) within the indoor experiment for *L. minor* ponds and also for the control ponds compared to the corresponding outdoor one. However, for the planted ponds comprising BR46 and the planted ponds fed with tap water mixed with fertiliser, no significant difference was noted (*p* > 0.05) between the two environmental conditions, which were 54 and 67 Pt-Co for ponds comprising BR46, and 44 and 46 Pt-Co for ponds containing tap water for indoor and outdoor experiments, respectively.

**Fig. 3:**
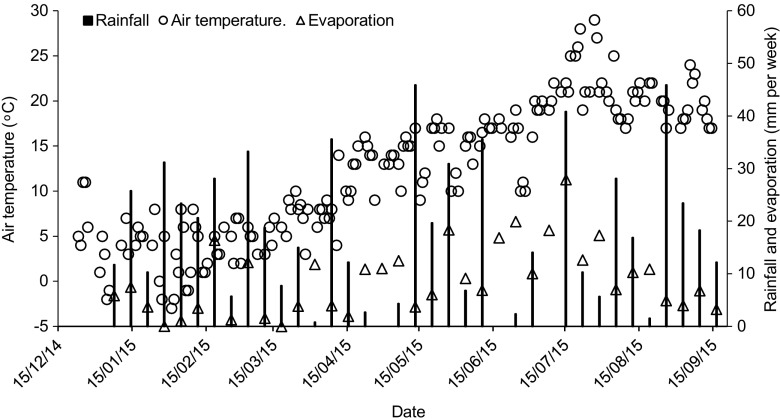
Environmental conditions for the outdoor experiment between 15 December 2014 and 15 September 2015

These findings regarding lower outflow values for the dye and colour under semi-natural conditions than the controlled ones can be discussed by the effect of rainfall under semi-natural conditions, which dilutes and subsequently reduces the content of these parameters in the ponds. Figure 3 indicates environmental boundary conditions such as rainfall in Salford during the study period. In addition, the process of removing excess water contributes to the reduction of the load of these parameters. Whereas under control conditions, there is no excess water requiring removal.

### Electrical conductivity and total dissolved solids

Mean outflow values for the electric conductivity (salinity indicator) were significantly higher (*p* < 0.05) for the laboratory compared to the semi-natural conditions (Fig. 2c), because of the dilution factor by rain and removing the excess water to achieve the required level under real conditions, which led to a reduction of the EC in the system. The mean outflow values were higher than the mean inflow ones for indoor experiments and slightly lower than the mean inflow values for the outdoor ones. This indicates low percentage reductions in EC values under controlled conditions. Moreover, all mean outflow values in the control ponds were elevated compared to the planted ponds, which denotes that the plants were able to reduce EC. The minimum and maximum mean outflow values ranged between 168 μS/cm in planted ponds comprising BR46 and 488 μS/cm in the control ponds containing RB198 under laboratory conditions, and between 78 μS/cm in *L. minor* ponds comprising BR46 and 102 μS/cm for control ponds containing RB198 under semi-natural conditions.

The mean total dissolved solid (ion concentration) values were half the EC values in this study, and all the inflow and outflow TDS values showed the same trend of the inflow and outflow EC values in terms of the indoor and outdoor experiments (Fig. 2d). These results match the findings by Amankwaah et al. (2014) who interpreted that EC is a function of TDS. These authors focused on the impact of pond effluents on the Asuofia Stream (Ghana) and found that the high values of EC could be attributed to the high TDS concentrations. All TDS outflow values for the indoor and outdoor experiments complied with the European and international standards of 2000 mg/l for discharge directly to receiving freshwater bodies (Carmen and Daniela 2012).

### Chemical oxygen demand and dissolved oxygen

The mean outflow COD concentrations (Fig. 2e) were higher within the indoor experiment than the corresponding values for the outdoor one. This was due to the rainfall dilution factor for outdoor experiments as discussed above. However, significantly (*p* < 0.05) higher differences were noted in planted ponds containing AB113, RB198 and control pond comprising tap water with fertiliser. In addition, all average outflow values were higher compared to the inflow ones, which indicates that the COD concentrations increased with time after each dose for both environmental conditions. This is due to the low COD removal efficiency, and also because some of the plants die off during their life cycle and increase the organic load in the ponds, which consequently rise the COD concentrations as discussed by Dalu and Ndamba (2003).

The international standards set a threshold concentration for COD of 125 mg/l concerning discharge of effluent directly into freshwaters (Carmen and Daniela 2012). The findings have indicated that the COD outflow concentrations were five, three, three and five times non-compliant for the planted and unplanted ponds containing AB113, control ponds containing BR46 and control pond comprising tap water mixed with fertiliser for the indoor experiment. However, for outdoor experiments, all COD values were below the set threshold.

Regarding the dissolved oxygen (DO) concentrations, the mean outflow values were higher than the mean inflow values for outdoor experiments, and slightly lower than the inflow for the indoor ones. The mean values of the outflow DO in planted and unplanted ponds were significantly (*p* < 0.05) higher within the outdoor experiment than those corresponding to the indoor one for all dyes, as well as the ponds containing tap water mixed with fertiliser (Fig. 2f). The minimum and maximum values ranged between 7.9 and 10.1 mg/l, and between 8.5 and 13.9 mg/l for the indoor and outdoor experiments, respectively. This is because the DO is highly affected by temperature variations; colder conditions increase the DO level and vice versa. Note that the temperature records for the outdoor experiment show wide variations compared with the controlled laboratory temperatures as mentioned previously. The presence of plants also leads to an increase in the DO level in the system due to respiration activities. However, in these experiments, it seems that temperature and oxygen diffusion by the atmosphere impacted on the DO values rather than the plants.

### pH and redox potential

The mean pH outflow values ranged between 7.3 and 8.4 for indoor experiments, and between 7.7 and 8.2 for the outdoor one (Fig. 2g). The mean outflow pH values for the *L. minor* and control ponds were higher within the outdoor experiment than the corresponding values within the indoor ones for all dyes except the control ponds treating BR46. Significant differences (*p* < 0.05) between the indoor and the outdoor experiment were found within the planted ponds only. However, the values were lower under semi-natural conditions than the resembling values under controlled conditions for *L. minor* and control ponds comprising tap water mixed with fertiliser, and control ponds treating BR46. Significant (*p* < 0.05) differences were only recorded for control ponds comprising tap water and fertiliser.

According to international standards (Carmen and Daniela 2012) for the discharge of effluents directly to receiving freshwater bodies, the acceptable pH limits were between 6.5 and 8.5. The pH outflow samples were 9 and 3, 4 and 6, 4 and 1, and 6 and 7, and 1 and 8 times non-compliant for systems containing AB113, Rb198, BR46, DO46 and tap water mixed with fertiliser for planted ponds and control ponds for outdoor experiments in that order. However, for indoor ones, only control ponds containing BR46, and tap water mixed with fertiliser, were 2 and 11 times not compliant.

Regarding the average values of the outflow redox potential, all values were lower than the inflow for the indoor and outdoor set-ups as shown in Fig. 2h. Higher mean outflow redox potential values were found for *L. minor* and control ponds regarding systems comprising AB113, RB198 and DO46, and planted ponds containing BR46 concerning the indoor experiment compared to the corresponding outdoor ones. Significant (*p* < 0.05) differences were found for planted ponds only. However, the values were lower within the indoor than the outdoor experiment for ponds with *L. minor*, control pond comprising tap water mixed with fertiliser and control ponds treating BR46. Significant (*p* < 0.05) difference was only noted for control ponds comprising tap water mixed with fertiliser.

### Suspended solids and turbidity

The mean suspended solids (SS) outflows for the indoor experiment were lower than the corresponding values for the outdoor one regarding all ponds except for control ponds comprising BR46, and control pond with tap water mixed with fertiliser (Fig. 2i). This could be due to the effect of natural conditions such as dust and rain for the outside experiment. Statistically, only the outflow values for planted ponds treating RB198 were significantly (*p* < 0.05) reduced within the indoor experiment compared with the outdoor one. However, outflow SS values for the control ponds containing BR46, and control ponds with tap water mixed with fertiliser, were significantly higher (*p* < 0.05) within the indoor experiment than the corresponding values for the outdoor one. The minimum and maximum values for control ponds containing BR46, and ponds with tap water mixed with fertiliser were 3 and 390 mg/l, and 2 and 268 mg/l, respectively. The main factors influencing SS removal in waste stabilisation ponds are biodegradation of organic matter, algal growth and sedimentation of particles (Dalu and Ndamba 2003). In this study, sedimentation and organic matter degradation affected the outflow SS concentrations.

The international standards for SS are 35 mg/l in case of discharges directly into watercourses. The samples were 13 and 11, 2 and 1, 20 and 17, 5 and 30 times non-compliant for planted and unplanted ponds containing AB113, Rb198 and BR46, planted ponds comprising DO46 and the control ponds comprising tap water mixed with fertiliser, respectively. However, for indoor experiments, the values were 9, 1, 3, 22 and 29 times non-compliant for planted ponds comprising AB113, unplanted ponds comprising AB113, planted ponds treating RB198, control ponds containing BR46 and control ponds containing tap water mixed with fertiliser, respectively.

Turbidity is a simple indicator for the clarity of water. For mean turbidity outflow values (Fig. 2j), no remarkable differences were noted between the indoor and the outdoor values except for the control ponds comprising BR46, DO46 and tap water mixed with fertiliser, which were significantly higher (*p* < 0.05) under controlled than natural conditions. Whereas, planted ponds comprising BR46 have shown to be significantly (*p* < 0.05) lower in terms of their mean turbidity outflows under controlled than natural conditions.

### Heavy metals and trace elements

Supplementary Fig. 1 shows an overview of the mean inflow and outflow concentrations of the nutrients and elements, which has been detected during the ICP-OES analyses for the indoor and outdoor experiments. Comparing the average values of inflow for the outdoor experiment with those for the indoor one, no significant (*p* < 0.05) differences were noted between the mean values for all noticed nutrients and elements between the two experiments. Further information can be found in the Supplementary Material 1.

### Dye removal

**Fig. 4:**
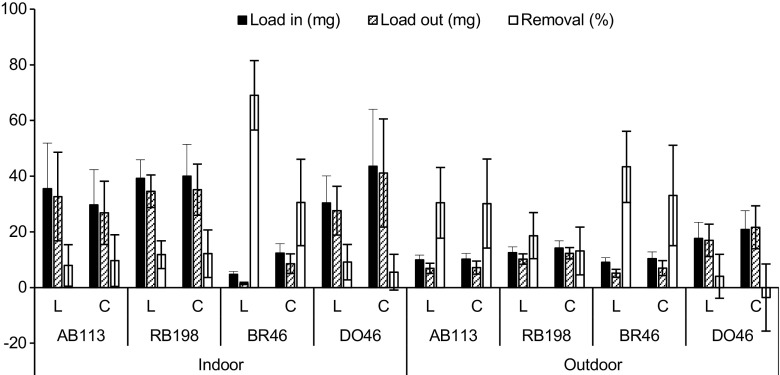
Mean and standard deviation of dye removal efficiency within the period between 15 December 2014 and 15 September 2015 for both experiments (*AB113* Acid blue 113, *RB198* reactive blue 198, *BR46* Basic Red 46, *DO46* Direct Orange 46, *L Lemna minor* ponds, *C* control ponds)

The dye removal efficiency within the period between 15 December 2014 and 15 September 2015 is shown in Fig. 4 for both experiments. AB113 removal for the outdoor experiment was significantly (*p* < 0.05) elevated than the indoor one for both planted and unplanted ponds. In addition, the mean removal efficiency for the planted and unplanted ponds was similar (both at around 30%) within the outdoor experiment, and very low (8 and 10%, respectively) for the indoor experiment. This indicates that *L. minor* was unable to break down AB113 molecules under controlled or semi-natural conditions, and the outdoor removal was due to the dilution effect of rainwater. In contrast, Balarak et al. (2016) reported on high removal efficiencies for AB113 using dried *L. minor.* This might be due to the pH of the solution adjusted to three, which enhanced the adsorption ability of the plants. However, in this study, the pH values were without adjustment, because the tolerated pH levels for growth of living *L minor* ranged between four and nine.

Although RB198 removal was small under both conditions, the mean removal efficiency for planted ponds was significantly (*p* < 0.05) elevated for the outdoor (19%) experiment than the indoor (12%) one. However, for the control ponds, no significant (*p* < 0.05) differences were noted.

The mean removal efficiency of BR46 in planted ponds was significantly (*p* < 0.05) reduced for outdoor experiments (43%) than indoor ones (69%). In addition, the control ponds were able to remove around 33 and 31% (no significant for *p* > 0.05) under semi-natural and controlled conditions, respectively. BR46 was treated better than other dyes in both experiments. The outflow samples were colourless, which is key evidence for the dye having been removed. In addition, during the study period, the maximum value of BR46 removal in planted ponds was 88 and 86% for the indoor and outdoor experiments, respectively. The impact of *L. minor* in terms of removal efficiency was around 38 and 10% under controlled and semi-natural conditions, respectively, (Fig. 4). This indicates that the high temperature under control conditions increases the removal efficiency of BR46. This result is matched by the findings reported by Khataee et al. (2012) suggesting that the biological removal efficiency of dyes improves with increased temperature as an endothermic process. The mean removal efficiency of DO46 was significantly (*p* < 0.05) higher within the indoor experiment than the outdoor one for planted and unplanted ponds. However, this dye exhibited low removal efficiency among the other dyes. Overall, statistical analysis showed that the dye BR46 was removed significantly (*p* < 0.05) greater than the other dyes for planted and unplanted ponds within the indoor system. However, for the outdoor experiment, the planted ponds showed significant (*p* < 0.05) higher removal efficiencies compared to the other dyes.

In general, the dye BR46 was treated better than the other dyes for both conditions. This may be due to the fact that BR46 is characterised by a simple chemical structure, small molecular weight and absence of sulphonic groups in their structure. In addition, the neutral pH was suitable for BR46 uptake. All these factors enhance the dye removal efficiency as discussed previously (Yaseen and Scholz 2016). However, it is worthy to note that the dyes DO46, AB113 and RB198 contained one, two and four sulpho groups in their structure, respectively, which may inhibit the degradation efficiency.

### Chemical oxygen demand removal

**Fig. 5:**
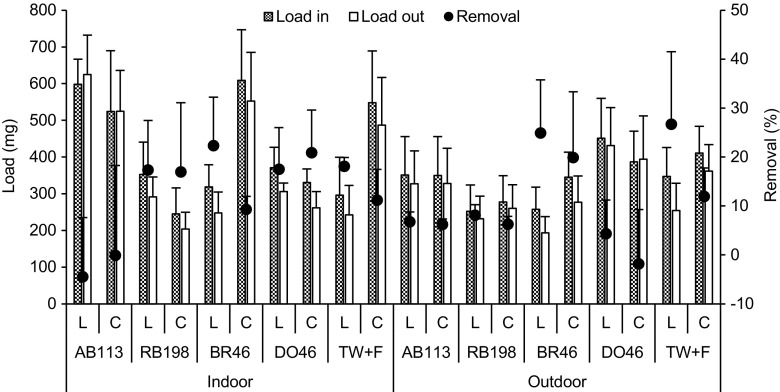
Mean and standard deviation of chemical oxygen demand removal efficiency within the period between 15 December 2014 and 15 September 2015 for both experiments (*AB113* Acid blue 113, *RB198* reactive blue 198, *BR46* Basic Red 46, *DO46* Direct Orange 46, *L Lemna minor* ponds, *C* control ponds)

Based on COD removal efficiencies, better removal was noted in planted and unplanted ponds containing AB113, BR46 and tap water mixed with fertiliser within the outdoor experiments compared to the indoor ones. However, lower removal efficiencies were noted within the outdoor experiment for planted and unplanted ponds containing RB198 and DO46. Significant differences (*p* < 0.05) were found for planted ponds comprising AB113 and planted and unplanted ponds containing DO46. Nonetheless, all COD removal values, which ranged between −4 and 22%, and −2 and 27% for the indoor and outdoor experiments, respectively, exhibited low microbial activities concerning COD mineralisation in these ponds (Fig. 5). This is evident indirectly by the high DO outflow concentrations. In addition, dead plants increase the organic load in the system and consequently increase the outflow COD concentrations as explained by Dalu and Ndamba (2003).

### Plant monitoring

The relative growth rate is normally applied as an indicator for the toxic impacts of dyes on the plant. In this study, the relative growth rate of *L. minor* based on the fresh weight (Movafeghi et al. 2013) was calculated after the experiment was finished and all plants in the ponds were harvested on 15 September 2015 for the indoor experiment, and on 2 February 2016 for the outdoor one (the outdoor experiment was completely finished).

In winter, *L. minor* is dormant, and individual plants settle down to the bottom of the pond system. The plant harvest included all dead, dormant and live plant parts found in the systems. Note that no additional plants were added to the system after the set-up.

**Fig. 6:**
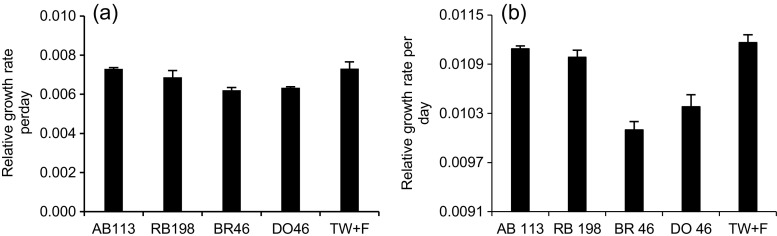
Mean and standard deviation of *Lemna minor* relative growth rate during the experimental operation period (**a** under semi-natural conditions, **b** under controlled conditions; *AB113* Acid blue 113, *RB198* reactive blue 198, *BR46* Basic Red 46, *DO46* Direct Orange 46, *TW+F* tap water and fertiliser)

The growth under controlled conditions was clearly higher than the one for the outdoor one due to the impact of environmental conditions in the laboratory (Table 1), which were suitable for optimum growth. In addition, Fig. 6a, b clearly shows that the lowest growth rate values were noted in ponds containing BR46 for both experiments. This clearly indicates that the BR46, which was treated, inhibited the growth of *L. minor*. Furthermore, the plants in tap water and fertiliser ponds showed growth rates that were higher compared to other ponds. This led to the presence of the dye in the system having a negative impact on the plant growth. Similar results were documented by Khataee et al. (2012).

## Conclusions

This study confirmed that the pond system planted with *L minor* was able (as a polishing step) to remove the dye BR46 at low concentrations from artificial wastewater significantly (*p* < 0.05) for both studied environmental conditions. However, the performance of these ponds under controlled conditions outperformed the ponds under semi-natural conditions by 23% concerning the BR46 removal efficiency. In addition, all outflow values concerning pH, TDS, COD and SS for the planted ponds containing BR46 under laboratory conditions were within the standard limits for direct discharge. This result suggests the suitability of operating the pond system in warmer regions. The outflow values of Zn and Cu were below the thresholds set and within the tolerated limits for plants in both experiments. The presence of the dyes inhibited the optimum growth of *L minor*, especially the treated dye BR46.

## Electronic supplementary material

ESM 1 (DOCX 121 kb)
