# Supplementary material for: Comparison of experimental ponds for the treatment of dye wastewater under controlled and semi-natural conditions
Source: Environ Sci Pollut Res Int. 2017 May 23;24(19):16031–40. doi: 10.1007/s11356-017-9245-5 (PMC5506514; doi:10.1007/s11356-017-9245-5)
Supplement: Supplementary file 1 — (DOCX 121 kb) [file 11356_2017_9245_MOESM1_ESM.docx]

#### **Supplementary Material 1**

#### **Comparison of experimental ponds for the treatment of dye wastewater under controlled and semi-natural conditions**

**Dina A. Yaseen^1^ • Miklas Scholz^1,2,3^**

Corresponding author: Miklas Scholz

miklas.scholz@tvrl.lth.se

Division of Water Resources Engineering, Department of Building and Environmental Technology, Faculty of Engineering, Lund University, P.O. Box 118, 22100 Lund, Sweden.

Tel.: +46 46 2228920; fax: +46 46 222 4435

^1^Civil Engineering Research Group, School of Computing, Science and Engineering, The University of Salford, Newton Building, Greater Manchester M5 4WT, UK

^2^Division of Water Resources Engineering, Department of Building and Environmental Technology, Faculty of Engineering, Lund University, P.O. Box 118, 22100 Lund, Sweden

^3^ Department of Civil Engineering Science, School of Civil Engineering and the Built Environment, University of Johannesburg, Kingsway Campus, PO Box 524, Aukland Park 2006, Johannesburg, South Africa

As shown in Supplementary Fig. 1a, the mean zinc outflow concentrations for *L. minor* and control ponds were significantly (p <0.05) higher within the indoor experiment than the outdoor one for ponds with and without dye. For outdoor experiment, the mean outflow zinc concentrations were less than the inflow one due to the rain factor. However, the indoor experiment showed that the mean outflow values were higher than the inflow ones except for the *L. minor* ponds treating BR46, which was slightly lower than the inflow. This might be due to the high degradation of this dye. For both experiments, the planted ponds showed mean outflow values slightly lower than the outflows of the corresponding control ponds due to the ability of *L. minor* in up-taking of zinc. Overall, for the two experiments, the mean outflow of zinc concentrations was lower than the threshold of 3 mg/l set for drinking water (Sasmaz et al. 2015). In addition, all outflow values were within the acceptable limits for *L. minor*. Zinc was tolerated by *L. minor* at 15 mg/l without any noticeable toxicity, but a reduction in the plant production rate of between 0.5 and 15 mg/l was reported by Khellaf and Zerdaoui (2009). Obvious damage to duckweed at a zinc concentration of 18 mg/l was reported by Khellaf et al. (2008). Therefore, in this study, zinc concentrations in the system did not impact negatively on the plant growth.

Regarding the mean outflow concentrations of copper, all values were significantly (p < 0.05) higher within the indoor experiment than the outdoor one (Fig. 4b). For the outdoor experiment, the mean outflow copper concentrations were lower than the inflow one, and the differences between the values for planted and unplanted ponds was very low. However, within the indoor experiment, the mean outflow values were higher than the inflow ones except for the planted ponds treating BR46. The planted ponds showed mean outflow values lower than the outflow concentrations of the corresponding control ponds indicating that the plants accumulate copper in their tissue, under laboratory conditions. Copper content was below the threshold limit of 2 mg/l for drinking water (Sasmaz et al. 2015). In addition, all outflow values were less than the tolerance limit of 0.4 mg/l for *L. minor* (Khaellaf and Zerdaou 2009) Khaellaf et al (2008) concluded that copper is a very toxic metal impacting on duckweed at a concentration of 0.5 mg/l. The copper levels in the systems had no adverse effects on the plant growth in this study.

Iron mean outflow concentrations (Supplementary Fig. 4c) for indoor experiments were significantly (p < 0.05) higher than those for the outdoor ones. For both indoor and outdoor set-ups, the mean outflow values were lower than the inflow ones except for the ponds containing AB113, which may effect on the iron content in the system. In addition, all planted ponds showed iron level less than the control ponds which indicated that the plants are responsible for the reduction.

The mean outflow concentrations of boron and potassium for the outdoor experiment were significantly (p < 0.05) lower than the ones for the indoor experiment (Supplementary Figs. 4d, e), respectively. Furthermore, the average outflow values for the outdoor set-up were lower than the inflow ones with very low differences between the planted and unplanted ponds. However, under controlled conditions, the mean outflow concentrations were higher than the inflow ones within the control ponds and lower than the inflow ones for the *L. minor* ponds.

Sodium mean outflow concentrations (Supplementary Fig. 4f) were significantly (p < 0.05) reduced within the outdoor experiment than the indoor one, and all outflow values were higher compared with the inflow concentrations. The indoor set-up shows that the mean outflow values were elevated within the control ponds than the *L. minor* ponds. However, the outdoor set-up shows that the mean outflow values were lower within the control ponds compared to the *L. minor* ponds. This may be because of the decay of the plants, which consequently increase the sodium content.

**Supplementary Fig. 1** Overview of the mean and standard deviation of inflow and outflow values for the detected trace elements and heavy metals within the indoor and outdoor experiments (AB113, acid blue 113; RB198, reactive blue 198; BR46, basic red 46; DO46, direct orange 46; TW+F, tap water and fertiliser; a, zinc; b, copper; c, iron; d, boron; e, potassium; f, sodium; g, calcium; h, magnesium).

The mean outflow concentrations for the calcium and magnesium concerning the outdoor experiment were significantly (p < 0.05) lower than the indoor one (Supplementary Figs. 4g, h), respectively. Under controlled conditions, the mean outflow values of these nutrients were higher than the inflow ones and the concentrations of planted ponds were lower than the control ones, indicating that the plants slightly reduce the concentrations to support growth. The findings matched those by Patel and Kanungo (2010) indicating that *L. minor* reduces the calcium and magnesium concentrations by 15% and 20%, respectively. However, under semi-natural conditions, the mean outflow values were lower than the inflow and the concentrations of planted ponds were higher than the control, indicating that the dead *L. minor* increased the content of the calcium and magnesium.

**Additional References**

Khellaf N, Zerdaoui M (2009) Growth response of the duckweed, *Lemna minor* to heavy metal pollution. J Environ Health Sci Eng 6:161–166

Khellaf N, Zerdaoui M, Faure O, Leclere J (2008) Tolerance to heavy metal in duckweed, *Lemna minor*. Environm Int 34:1022–1026

Patel D, Kanugo V (2010) Pyhtoremediation potential of duckweed (*Lemna minor* L: a tiny aquatic plant) in the removal of pollutants from domestic wastewater with special reference to nutrients. Bioscan Int Quart J Life Sci 5:355–358

Sasmaz M, Topal E, Obek E, Sasmaz A (2015) The potential of Lemna gibba L. and Lemna minor L. to remove Cu, Pb, Zn, and As in gallery water in a mining area in Keban, Turkey. J Environm Managem 163:246–253.
